# Differential requirements for cytokine signaling and local antigen in upper versus lower respiratory tract CD8^+^ T_RM_ development

**DOI:** 10.1093/jimmun/vkag269

**Published:** 2026-10-17

**Authors:** Kirsten K Rodgers, Mark Elliott Williams, Ariana R Jimenez, Sakeenah L Hicks, Cameron L R Mattingly, Sarah E Michalets, Yixel M Soto-Vázquez, Kathryn M Moore, Madeleine M Rasche, Laurel A Lawrence, Yu-shan Huang, Thien Duy Chen, Sofia R Cendoya, Christopher D Scharer, Alison Swaims-Kohlmeier, Jacob E Kohlmeier

**Affiliations:** Department of Microbiology and Immunology, Emory University School of Medicine, Atlanta, GA, United States; Department of Microbiology and Immunology, Emory University School of Medicine, Atlanta, GA, United States; Department of Microbiology and Immunology, Emory University School of Medicine, Atlanta, GA, United States; Department of Microbiology and Immunology, Emory University School of Medicine, Atlanta, GA, United States; Department of Microbiology and Immunology, Emory University School of Medicine, Atlanta, GA, United States; Department of Microbiology and Immunology, Emory University School of Medicine, Atlanta, GA, United States; Department of Microbiology and Immunology, Emory University School of Medicine, Atlanta, GA, United States; Department of Microbiology and Immunology, Emory University School of Medicine, Atlanta, GA, United States; Department of Microbiology and Immunology, Emory University School of Medicine, Atlanta, GA, United States; Department of Microbiology and Immunology, Emory University School of Medicine, Atlanta, GA, United States; Department of Microbiology and Immunology, Emory University School of Medicine, Atlanta, GA, United States; Department of Microbiology and Immunology, Emory University School of Medicine, Atlanta, GA, United States; Department of Microbiology and Immunology, Emory University School of Medicine, Atlanta, GA, United States; Department of Microbiology and Immunology, Emory University School of Medicine, Atlanta, GA, United States; Department of Microbiology and Immunology, Emory University School of Medicine, Atlanta, GA, United States; Department of Gynecology and Obstetrics, Emory University School of Medicine, Atlanta, GA, United States; Department of Microbiology and Immunology, Emory University School of Medicine, Atlanta, GA, United States

**Keywords:** cytokines, influenza, respiratory tract, tissue-resident memory T cells

## Abstract

CD8^+^ tissue-resident memory T cells (T_RM_) in the upper respiratory tract (URT) limit viral replication after transmission and prevent spread to the lower respiratory tract (LRT), whereas LRT CD8^+^ T_RM_ are important for limiting immunopathology in the lung and promoting efficient viral clearance. Although the presence of local respiratory antigen is thought to be required for CD8^+^ T_RM_ development in the LRT but not the URT, the relationship between local antigen encounter and cytokine signaling that influences T_RM_ differentiation in distinct niches of the respiratory tract remains unknown. Here, we show using murine models that while URT T_RM_ generated in the presence or absence of local respiratory antigen are phenotypically, transcriptionally, and functionally similar throughout the respiratory tract, their developmental pathways are compartmentally distinct and characterized by a context-dependent compensatory relationship between antigen and cytokine signaling. In the absence of local antigen, URT CD8^+^ T_RM_ formation required TGF-β signaling, and notably, while both IL-15 and IL-33 were broadly dispensable in the URT, IL-33 was able to rescue LRT CD8^+^ T_RM_ development in the absence of local antigen. Together, these findings highlight key differences for CD8^+^ T_RM_ development in distinct areas of the respiratory tract, offering valuable insights into the development of mucosal cellular immunity following intranasal and parenteral vaccination.

## Introduction

Respiratory viruses such as influenza, respiratory syncytial virus (RSV), and severe acute respiratory syndrome coronavirus 2 (SARS-CoV-2) impose a substantial burden on human health across the globe, with influenza A virus infections resulting in hundreds of thousands of deaths annually.^1^ Respiratory viruses are initially seeded in the upper respiratory tract (URT), and if infection is controlled at this site, the outcomes are likely to be mild to moderate clinical symptoms and rapid viral clearance.^2^ Effective clearance is a multi-faceted process involving innate immune responses that slow viral propagation, as well as virus-specific antibodies and T cell responses that restrict spread and eliminate infected cells, respectively. However, when immunity is suboptimal, delayed clearance in the URT can permit viral dissemination to the lower respiratory tract (LRT), leading to pneumonia and other severe clinical complications.^3–7^ Therefore, defining the immune mechanisms that promote viral clearance across different regions of the respiratory tract, while preventing immunopathology, are critical for reducing disease burden.

Tissue-resident memory T cells (T_RM_) play a critical role in limiting pathogen replication during the initial stages of infection due to their proximity with mucosal surfaces and their rapid induction of effector functions.^8–10^ In the respiratory tract, CD8^+^ T_RM_ have been shown to provide to robust heterosubtypic protection against influenza virus^11–16^ and are associated with reduced viral burden and better clinical outcomes to SARS-CoV-2 and RSV infections.^17–19^ Although present throughout the respiratory tract, different populations of CD8^+^ T_RM_ provide unique contributions to protection against severe disease, with URT CD8^+^ T_RM_ being essential for preventing viral spread to the lung and LRT CD8^+^ T_RM_ being crucial for efficient viral clearance in the lung while limiting immunopathology.^20–22^ Due to their importance for immunity, many studies have investigated the cellular interactions and molecular cues that guide T_RM_ development in the respiratory tract.^23–25^ For example, IL-15 is known to be important for memory T cell survival and has been implicated in modulating effector functions of memory T cells.^26^ TGF-β promotes upregulation of the residency marker CD103 and adoption of a T_RM_ transcriptional program in various mucosal sites, including the lung.^27–29^ IL-33 signaling has similarly been shown to have a role in respiratory tract T_RM_ development by working in concert with TGF-β signaling to downregulate Klf2, a transcription factor that promotes a circulatory T cell program.^30^ However, it was previously shown that URT T_RM_, but not LRT T_RM_, can form in the absence of TGF-β. Furthermore, several studies in mice and humans have demonstrated that formation of LRT CD8^+^ T_RM_ requires localized respiratory antigen, in contrast to URT CD8^+^ T_RM_.^31–33^ Together, these findings provide evidence that the requirements for T_RM_ formation differ between these unique niches of the respiratory tract.

Many prior studies investigating respiratory tract CD8^+^ T_RM_ development have utilized intranasal virus infections, in which localized respiratory antigen is present. However, given that many vaccines targeting respiratory viruses are delivered via non-respiratory routes and fail to deposit antigen to the respiratory tract, it is critical to understand how the presence or absence of local respiratory antigen alters the cytokine requirements for T_RM_ development in different respiratory tract compartments. Here, we utilized a murine influenza virus infection model to examine URT and LRT CD8^+^ T_RM_ development in both the presence and absence of local antigen. We show that URT CD8^+^ T_RM_ formed under different antigenic conditions are phenotypically and functionally similar and that in both cases CD8^+^ T_RM_ established similar localization patterns in nasal cavity tissue. In addition, we found that URT CD8^+^ T_RM_ have a nearly identical transcriptional program regardless of local antigen but are transcriptionally distinct from LRT CD8^+^ T_RM,_ despite showing a high degree of clonal overlap. Using cytokine receptor conditional knockout mice, we found that IL-33 and IL-15 signaling were dispensable for CD8^+^ T_RM_ development in both the URT and LRT regardless of local respiratory antigen. However, we observed that while TGF-β signaling contributes to URT CD8^+^ T_RM_ development in the presence of local respiratory antigen, TGF-β became essential for URT T_RM_ formation in the absence of antigen. In contrast, TGF-β was required for LRT T_RM_ formation in the presence of local antigen. Finally, we demonstrated that IL-33 signaling can partially rescue LRT T_RM_ formation in the absence of local antigen. Together, our work defines the site-specific requirements for URT CD8^+^ T_RM_ development, distinct from that of LRT CD8^+^ T_RM,_ in both the presence and absence of local respiratory antigen.

## Materials and methods

### Mice, viruses, and treatments

Briefly, 6- to 8-wk-old female and male B6 CD45.2^+^ (000664), B6 CD45.1^+^ (Pep Boy) (002014), IL15Ra^fl^ (022365), Il1r^loxP^ (028398), TGFBR2^fl^ (012603), and E8ICre (008766) mice were acquired from Jackson Laboratory and housed under specific pathogen free (SPF) conditions at Emory University. Upon delivery from Jackson Laboratory, all mice were rested for one week upon arrival. Mice were maintained in a temperature-controlled room (72°F setpoint) with a relative humidity of 40%–50% and 12-hour light/dark cycle. Conditional knockout and wild-type control mice were generated by crossing IL15Ra^fl^, Il1r^loxP^, and TGFBR2^fl^ with E8ICre mice. For most experiments, 8- to 14-wk old female and male conditional knockout and wild-type control mice were utilized. For naïve (pre-infection) experiments, 6- to 8-wk-old female and male TGFBR2^fl^ E8I Cre (conditional knockout) and wild-type control mice were used. All animal protocols adhered to the National Institutes of Health (NIH) *Guide for the Care and Use of Laboratory Animals* and were approved by the institutional animal care and use committee at Emory University (PROTO201700581). For intranasal priming, 30,000 50% egg infectious dose (EID50) A/HKx31 (×31) was administered in a 30 µL volume under isoflurane anesthesia (Patterson Veterinary). For intraperitoneal priming mice were administered 3.2 × 10^6^ EID50 in 200 µL. For intramuscular priming mice were administered 3.2 × 10^6^ EID50 in 50 µL. For experiments utilizing recombinant mouse IL-33 (R&D Systems), 0.5 µg in 30 µL was administered intranasally at days 7 and 9 post-infection under isoflurane anesthesia.^34^ In addition, 5 µg ODN 1826 (CpG) (InvivoGen) in 30 µL was administered intranasally under isoflurane anesthesia in combination with IL-33 or alone as a control.

### Single cell isolation, staining, flow cytometry, and cell sorting

Prior to sacrifice, animals were intravenously (IV) labeled via tail vein injection with a fluorescent antibody, either 4 µg CD45.2-A700 clone 104 or 2 µg CD45.2-PE clone 104 in 200 µL phosphate buffered saline (PBS) for all experiments. IV label was permitted to circulate for 5 min, and mice were then euthanized by i.p. injection of avertin (2,2,2-tribromoethanol) followed by brachial exsanguination. Spleen, lungs, and nasal cavity tissue were then harvested into chilled R10 (RPMI 1640 (Corning), 10% fetal bovine serum HI (MedSupply partners), and 1% penicillin/streptomycin (Genesee Scientific)) or Hanks’ Balanced Salt Solution (Corning). Nasal cavity, lungs, and spleen were isolated and processed as previously described.^21^ In brief, tissue was mechanically digested (spleen, lungs, nasal cavity) followed by enzymatic digestion with DNAse I (Stem Cell Technologies) and collagenase D (Roche) (lungs, nasal cavity). Nasal cavity was carefully filtered through 70 µM strainers to remove any undigested fragments. For flow cytometry analysis, spleen and nasal cavity samples were ACK lysed. For fluorescence activated cell sorting (FACS) prior to RNA sequencing, CD8 isolation was performed with either CD8a^+^ T cell isolation kit, mouse (Miltenyi Biotec) or StraightFrom® Spleen CD8a^+^ T cell isolation kit, mouse (Miltenyi Biotec) per manufacturer’s instructions for optimal recovery. For flow cytometry and sorting, single cell suspensions were first incubated with murine Fc block (aCD16/32 clone 2.4G2) for 10 minutes on ice. Samples were then stained with Flu NP (D^b^_366-374_) tetramer or Flu PA (D^b^_224-233_) tetramer conjugated to PE or BV421 at a 1:100 dilution for 1 h in the dark at room temperature, followed by surface staining with various combinations of the following fluorescently conjugated antibodies at 1:100 dilution: CCR2, CD3e, CD4, CD8a, CD11b, CD11c, CD19, CD44, CD45, CD49a, CD62L, CD69, CD103, CD127, CXCR3, CXCR6, CX3CR1, KLRG1, Ly6C, Ly6G, MHC-II. For intracellular FluNP staining, fixation and permeabilization was performed utilizing a fix/perm kit (BD Biosciences) per manufacturer’s instructions, followed by intracellular staining with 1:20 dilution of FluNP-FITC clone D67J (Invitrogen). For both FACS and flow cytometry, viability was determined using a 1:200 dilution of Zombie NIR dye (Biolegend) or 7-AAD (Biolegend). Data was acquired on a FACSymphony A3 (BD Biosciences) flow cytometer or sorted on either a FACSAriaII (BD Biosciences) or Aurora (Cytek). Flow cytometry data analysis was conducted using FlowJo v.10 software. All antibodies were purchased from Biolegend or BD Biosciences and relevant tetramers were generously provided by the NIH tetramer core facility of Emory University in Atlanta, Georgia, USA.

### Peptide stimulations and intracellular cytokine staining

Single cell suspensions prepared from nasal cavity tissue as described above were stimulated with FluNP_366-374_ and FluPA_224-233_ peptides (New England Peptide LLC) for 6 h at 37 °C 5% CO2. PMA/Ionomcyin and media alone served as positive and negative controls, respectively. For intracellular cytokine staining (ICCS), brefeldin A (1 µL/mL) and monensin (4 µL/6 mL) from BD Biosciences were added to R10 media (described above) containing 10% β-mercaptoethanol prior to stimulation and for all surface and intracellular staining steps thereafter. For flow cytometry, cells were washed with PBS and stained with live/dead and extracellular stain as described above. Prior to stimulation, cells were split and some were used for tetramer staining, live/dead, and surface staining as described above. For ICCS, fixation and permeabilization was performed utilizing a fix/perm kit (BD Biosciences) per manufacturer’s instructions, followed by intracellular staining with 1:100 dilution of TNF-α:PE clone MP6-XT22 (Biolegend) and IFN-γ:APC clone XMG1.2 (BD Biosciences).

### Bulk RNA sequencing

For bulk RNA sequencing, 72–5,000 cells were sorted directly into RLT lysis buffer (Qiagen) containing 1% β-mercaptoethanol for RNA release and were subsequently flash frozen. Total RNA was isolated using the Quick-RNA Microprep kit (Zymo Research). Synthesis of cDNA was performed using SMART-seq v4 Ultra Low Input RNA kit (Takara). Final libraries were generated using 200 pg of cDNA as input for the NexteraXT kit (Illumina) with 12 cycles of PCR amplification. Final RNA-seq libraries were quantitated by QuBit (Life Technologies), size distributions determined by bioanalyzer (Agilent 2100), pooled at equimolar ratios, and sequenced at the EPC Genomics Core on a NovaSeq6000 using a PE100 run. Raw sequencing reads were mapped to the mm10 reference genome using STAR v2.7.11b,^35^ and duplicate reads were marked by MarkDuplicates (Picard) v3.1.1^36^ and removed before downstream analysis. The threshold level of detection for reads was set to 3 reads per million (RPM); all members of at least one phenotypic group meeting this threshold were kept, and reads below the threshold were discarded. Reads per kilobase per million (RPKM) normalized gene expression counts were derived by analyzing coverage across all exons that fall within unique ENTREZ genes using the tool GenomicRanges v1.54.1^37^ and custom R v4.1.0 scripts. DESeq2 v1.42.1^38^ was used to determine differentially expressed genes (DEG) which were defined as having an absolute log2 fold-change of ≥1 and a false discovery rate (FDR) of ≤0.05. The principal component analysis (PCA) plot in Fig. 2 was generated using only the union of all the significant genes from each group compared pairwise to the other groups using the tools som v0.3-5.1, dplyr v1.1.2, ggtext v0.1.2, and ggplot2 v3.4.2. Targeted Gene Set Enrichment Analysis (GSEA) for Fig. 2 was performed using the tool GSEA v4.3.2^39^^,^^40^ with specific gene list inputs (Table S1).

### Single cell RNA sequencing

Gene expression and TCR single cell RNA libraries of FACS sorted bulk, tissue-resident (lung, nasal cavity) or total bulk (spleen) FluNP and FluPA tetramer^+^ CD8^+^ T cells were generated with the Chromium Single Cell 5′ Reagent Kit v2 (dual index) (10X Genomics) per manufacturer’s instructions. For each sample, an estimated 524–21,000 cells were used for GEM and library generation. Library QC and sequencing were performed as described above. After sequencing, Illumina fastq files were processed and mapped to the GRCm39 (GENCODE vM33/Ensembl110 annotations) reference transcriptome using 10x Genomics Cell Ranger 8.0.0. For TCR sequencing, immunodominant Influenza NP and PA CD8 sequences from VDJdb were applied to sequencing reads.^41^ Biological replicates were aggregated for downstream analysis using the “aggr” command in Cell Ranger with the default parameters. Aggregated data were analyzed using Seurat v5.3.1.^42^ Low quality cells with the following criteria were filtered out: percentage of mitochondrial reads in each cell greater than 5%, less than 300 genes with reads per cell, and greater than 10,000 genes with reads per cell. The counts data were normalized first for library-size and then log transformed using the NormalizeData function in Seurat. Linear dimensional reduction was run using the variable features as input features with the RunPCA command. A cell similarity graph was generated using FindNeighbors command with the “dims” argument set to the first 40 principal component dimensions to identify transcriptomically similar clusters using the FindClusters command with the “resolution” argument set to 0.25 and the “algorithm” argument set to 3. Finally, non-linear dimensional reduction was performed using the RunUMAP command with the “reduction” argument set to “PCA” and “dims” argument set to the first 60 dimensions. For all data in Fig. 4, T_RM_ in lung and nasal cavity were defined as cells expressing *Itgae* at log10 normalized expression level of ≥0.5, and T_EM_ in spleen and non-T_RM_ in lung and nasal cavity were defined as all cells NOT expressing *Itgae* alone at a normalized expression level of ≥0.5, *Cd69* alone at a normalized expression level of ≥0.5, or both markers in combination at a normalized expression level of ≥0.5. DEGs were calculated using the FindAllMarkers command. Pathway analysis was performed using Single Cell Pathway Analysis v1.6.2.^43^

### Adoptive transfer and immunofluorescence microscopy

Briefly, 10,000 P14 CD45.2^+^ CD8^+^ T cells, isolated using CD8a^+^ T cell isolation kit, mouse (Miltenyi Biotec) were transferred intravenously to CD45.1^+^ recipients. Recipients were infected with x31-NAgp33 intranasally or intraperitoneally as described above. At resting memory (approximately 30 d post-infection), nasal cavity tissue was collected as previously described. Immediately after collection, samples were de-gassed for 5 min to remove air bubbles and fixed in 2.5% neutral buffered formalin for 3 h room temperature and then incubated overnight at 4 °C. Fixed tissue was then moved to 0.5 M EDTA solution for 5–7 d at 4 °C to decalcify bone. Tissue was embedded in OCT and flash-frozen. Blocks were sectioned at 20 microns on a cryostat and slides were fixed in 75:25 acetone/ethanol. Fixed slides were blocked with FACS buffer containing 1 µg/mL anti-mouse CD16/32 (clone 2.4G2) antibody, 10% normal rat serum, and 10% normal hamster serum. Staining was performed with anti-mouse CD45.2-A647 (clone 104 Biolegend), CD103-A594 (clone 2E7 Biolegend) and EPCAM-A488 (clone G8.8 Biolegend). Sections were mounted with mounting media containing DAPI (Abcam). Slides were cured for 24 to 72 h before imaging on a Zeiss Axio Observer Z1 immunofluorescence microscope with an Axiocam 506 monochromatic camera. Image processing was performed with Zen 2 software. T_RM_ were quantified via blinded scoring in tissue sections set to a common area of 1,000 µM^2^.

### Data analysis and statistics

Analyses were performed using GraphPad Prism software v10.6.1 or R. Methods for data comparisons and corrections are reported for each figure in the corresponding legend. As noted in the figure legends, a cutoff of 10 cells was used for data exclusion in flow cytometry experiments reporting absolute cell numbers. For flow cytometry experiments, a cutoff of 50 cells from the parent gate was utilized to confirm that staining was representative of the cell population.

## Results

### Upper respiratory tract CD8^+^ T_RM_ can develop in the absence of local respiratory antigen

To assess T_RM_ formation in the presence or absence of local respiratory antigen, we infected wild-type mice with influenza A-HK/x31 (x31) either intranasally (i.n.) or intraperitoneally (i.p.) and rested them for 30–35 d (Fig. 1A-C). We defined a dose for a non-respiratory (intraperitoneal) administration route that resulted in an equivalent number of circulating CD8^+^ influenza-specific memory T cells (Fig. 1D). Lung T_RM_ were detected after i.n. infection, but not i.p. infection (Fig. 1C and E), recapitulating previous findings that formation of lower respiratory tract (LRT) T_RM_ requires encounter with antigen in the local respiratory microenvironment (herein referred to as local respiratory antigen).^20^^,^^32^^,^^33^^,^^44^ In contrast, nasal cavity T_RM_ in the upper respiratory tract (URT) formed regardless of infection route, showing that local antigen is beneficial, but not required, for T_RM_ development in this site (Fig. 1C and E). A fold-change comparison of T_RM_ after i.n. versus i.p. priming further demonstrates that local antigen impacts LRT T_RM_ to a greater degree than URT T_RM_ (Fig. 1F). To confirm the absence of antigen in the local URT microenvironment after i.p. priming, we assessed the frequency of tissue-resident antigen-presenting cell subsets harboring intracellular influenza nucleoprotein (FluNP) during the peak of the effector phase (day 6 post-infection) (Fig. 1G). Sendai virus (SeV) infection was included as a control to confirm the specificity of FluNP staining.^45^ FluNP-harboring tissue-resident classical monocytes were observed in both the URT and LRT after i.n. x31 infection but not after i.p. x31 or i.n. SeV infection. (Fig. 1H and I). We observed a similar trend when assessing tissue-resident FluNP-harboring dendritic cells and macrophages (Fig. 1J and K). Similar to i.p. infection, administration via another common parenteral route, intramuscular, generated T_RM_ in the URT, but not the LRT (Fig. S1A to D). Together, these data show that, unlike LRT T_RM_, formation of URT T_RM_ can occur independently of local respiratory antigen.

**Figure 1:**
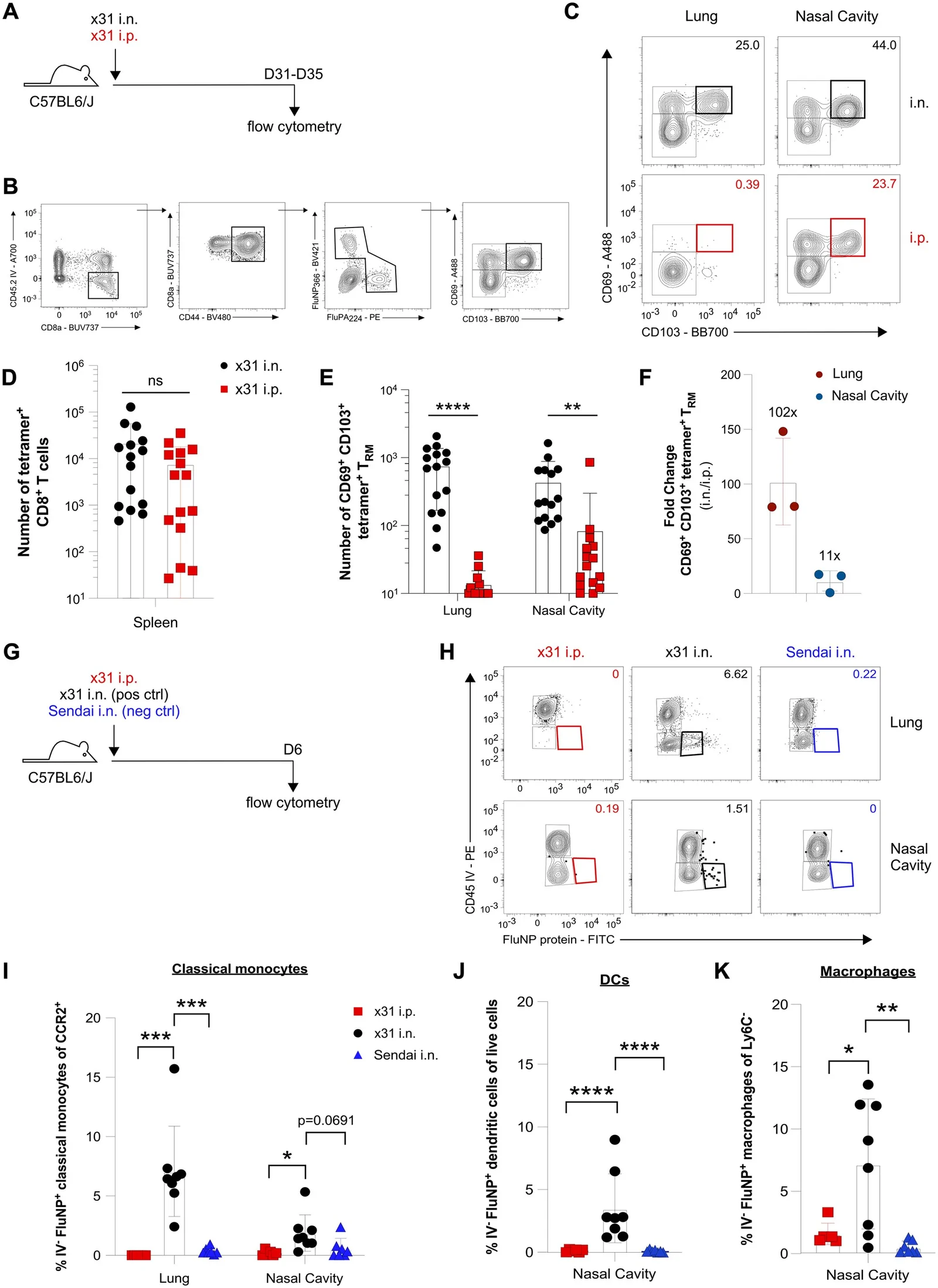
CD8^+^ T_RM_ develop in distinct respiratory sites in the presence or absence of local respiratory antigen. (A) Experimental setup to assess respiratory tract T_RM_ after i.n. or i.p. priming. (B) Representative gating for identification of influenza-specific T_RM_. (C) Representative staining for CD69 and CD103 on influenza-specific respiratory tract T_RM_ after i.n. or i.p. priming. (D) Absolute number of influenza-specific splenic CD8^+^ T cells after i.n. or i.p. priming. (E) Absolute number of URT and LRT influenza-specific T_RM_ after i.n. or i.p. priming. (F) Fold change in absolute number of URT and LRT influenza-specific T_RM_ after i.n. versus i.p. priming, calculated from average of *n* = 3 independent experiments. (G) Experimental setup to assess URT FluNP-harboring antigen presenting cell subsets after i.n. or i.p. priming. (H) Representative staining for LRT and URT FluNP-harboring tissue-resident classical monocytes after i.n. or i.p. priming. (I) Frequency of tissue-resident URT and LRT FluNP-harboring classical monocytes. Gated on CD45^+^ CD11c^−^ Ly6G^−^ CD11b^+^ CCR2^+^. (J) Frequency of tissue-resident URT FluNP-harboring dendritic cells. Gated on CD45^+^ MHC-II^+^ CD11c^+^. (K) Frequency of tissue-resident URT FluNP-harboring macrophages. Gated on CD45^+^ MHC^−^II^+^ CD11c^−^ Ly6G^−^ CD11b^+^ Ly6C^−^. Data represented as mean ± SD from *n* = 2–3 independent experiments with *n* = 6–15 mice per infection condition (D–E, I–K). Fold change representative of mean from 3 independent replicates with *n* = 3–5 per replicate (F). Absolute cell numbers utilize 10 cell minimum cutoff (D–E). Significance determined by unpaired T test (D–E, I–K). *P* values are as follows: NS 0.1234, *P 0.0332, **P 0.0021, ***P 0.0002, ****P < 0.0001.

### Development of T_RM_ and T_EM_ precursors within the nasal cavity is not altered by local respiratory antigen

In the early stages of respiratory virus infection, activation of CD8^+^ T cells gives rise to short lived effector cells (SLEC) and memory precursor effector cells (MPEC).^46^ The former perform effector functions to control viral spread and subsequently contract when antigen is fully cleared; the latter persist and give rise to the central, effector, and tissue-resident memory CD8^+^ T cell pools. While formation of MPEC and T cell pools is well characterized, less so is the role of local antigen for their development and expansion in respiratory tissue. Therefore, we next investigated whether local respiratory antigen was altering the dynamics of memory precursor cell development in virus-specific URT CD8^+^ T cells. Mice were infected with x31 i.n. or i.p., and the generation of influenza-specific memory precursor CD8^+^ T cells (CD127^+^ KLRG1^−^, MPEC) in the URT for both T_RM_ (CD69^+^ CD103^+^, MPEC T_RM_) and T_EM_ (CD69^−^ CD103^−^, MPEC T_EM_) was monitored over time (Fig. 2A and B). We included analysis of surface markers associated with T_RM_ (CXCR6, CXCR3, and CD49a) or T_EM_ (CX3CR1) trafficking (Fig. 2C). While expansion of URT T_RM_ precursors was delayed after i.p. priming compared to i.n. priming (Fig. 2F; Fig. S2A and B) both URT precursor pools showed similar developmental kinetics and expression of trafficking molecules regardless of infection route (Fig. 2D and E). However, URT T_RM_ precursors expanded to greater frequencies by day 14 and settled at a significantly higher frequency at memory (day 30) after i.n. compared to i.p. priming (Fig. S2C), consistent with earlier findings (Fig. 1E).

**Figure 2:**
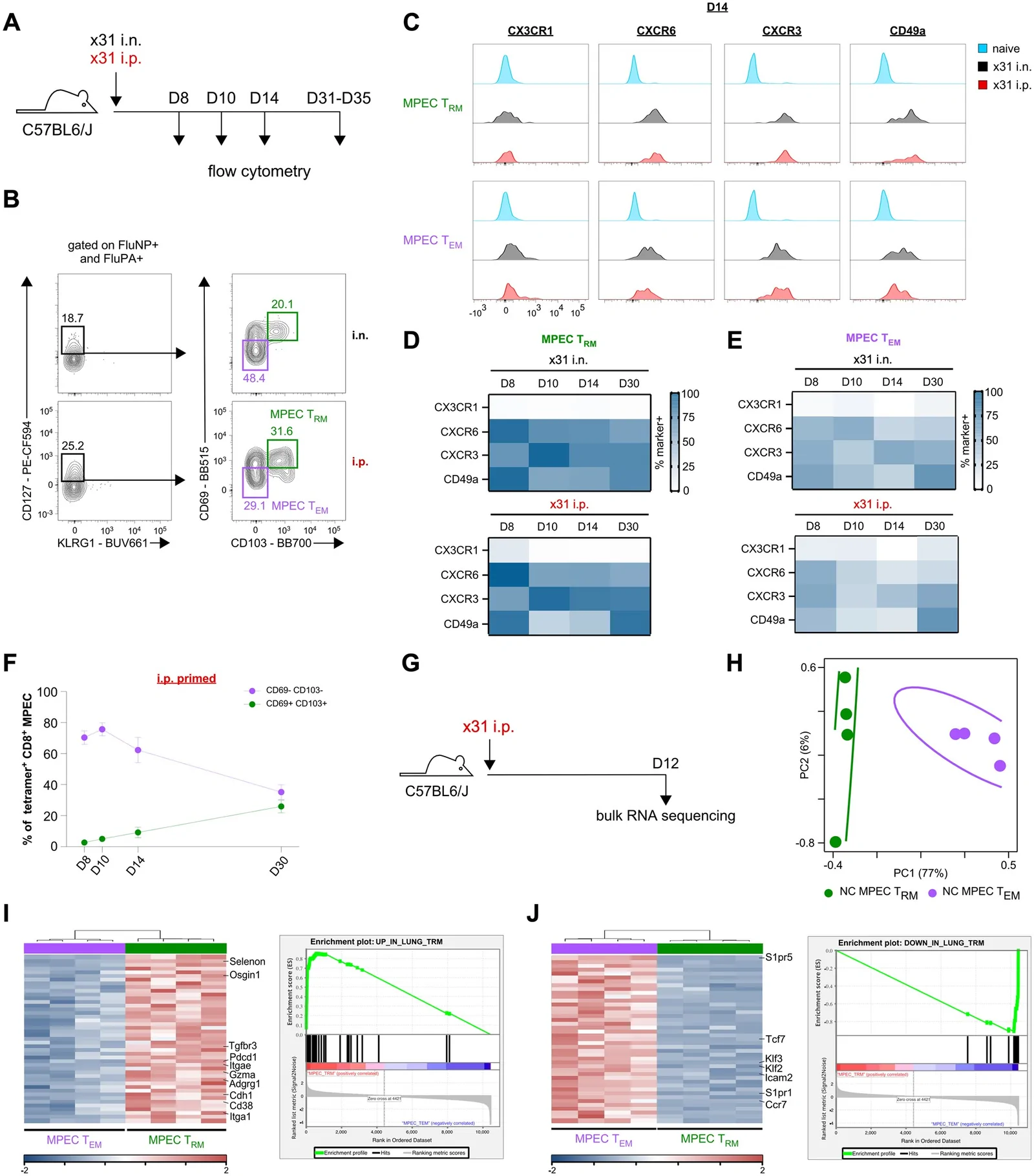
Distinct URT CD8^+^ T_RM_ and T_EM_ precursor pools form in the absence of local antigen. (A) Experimental setup to assess formation of URT memory precursor or bona fide T_RM_ and T_EM_ after i.n. and i.p. priming. (B) Representative gating (day 14 post-infection) for identification of influenza-specific memory precursor (CD127^+^ KLRG1^−^) (MPEC) T_RM_ (CD69^+^ CD103^+^) and T_EM_ (CD69^−^ CD103^−^) after i.n. or i.p. priming. (C) Representative histograms of T_RM_-associated marker expression on URT MPEC T_RM_ or T_EM_ after i.n. or i.p. priming. Naïve cells included as a staining control. (D) Heatmap illustrating frequency of URT MPEC T_RM_ or bona fide T_RM_ expressing T_RM_-associated markers after i.n. or i.p. priming. (E) Heatmap illustrating frequency of URT MPEC T_EM_ or bona fide T_EM_ expressing T_RM_-associated markers after i.n. or i.p. priming. (F) Frequency of URT MPEC T_EM_ and T_RM_ over time after i.p. priming. (G) Experimental setup to assess transcriptional profiles of URT MPEC T_RM_ after i.p. priming. (H) PCA plot of sorted MPEC T_EM_ and T_RM_ after i.p. priming. (I) Hierarchical clustering heatmap of bulk RNA transcript expression for indicated differentially expressed genes (left) and enrichment plot for targeted GSEA of genes with increased expression in T_RM_ (right). (J) Hierarchical clustering heatmap of bulk RNA transcript expression for the indicated differentially expressed genes (left) and enrichment plot for targeted GSEA of genes with decreased expression in T_RM_ (right). Data represented as mean ± SEM from *n* = 3 independent experiments with *n* = 3–15 mice per timepoint and infection condition (D–F). Bulk RNA sequencing data compiled from *n* = 4 replicates with 10 mice pooled per replicate (H–J).

To determine whether bona fide T_RM_ and T_EM_ transcriptional programs are imparted at the earliest stages of MPEC subset differentiation in the absence of respiratory antigen, we infected mice with x31 i.p. and sorted virus-specific CD8^+^ URT MPEC T_RM_ or T_EM_ at day 12 post-infection for bulk RNA sequencing (Fig. 2G). Principal component analysis (PCA) revealed clear separation in the transcriptional programs of URT MPEC T_RM_ and T_EM_ at this early stage of development (Fig. 2H). Analysis of differentially expressed genes (DEGs) between these subsets and targeted gene set enrichment analysis (GSEA) against a curated T_RM_ gene set (Table S1)^47^ revealed that URT MPEC T_RM_ were highly enriched for genes that were upregulated in tissue-resident cells, such as such as *Selenon*, *Osgin1*, *Pdcd1*, *Itgae*, *Gzma*, *Adgrg1*, *Cdh1*, *Cd38*, and *Itga1* (Fig. 2I). In contrast, URT MPEC T_EM_ were highly enriched for genes that were downregulated in tissue-resident cells, such as *S1pr5*, *Tcf7*, *Klf2*, *Klf3*, *Icam2*, *S1pr1*, and *Ccr7*^30^ (Fig. 2J). Together, these data demonstrate that the early bifurcation of the MPEC pool into T_RM_ and T_EM_ lineages in the URT occurs independently of local respiratory antigen.

### Local respiratory antigen does not alter the localization or functionality of URT CD8^+^ T_RM_

Given their proximity to the mucosal surface, respiratory tract T_RM_ can release potent effector cytokines that act directly on nearby epithelial cells to induce antiviral programming^48^ critical for protective immunity.^49^ Therefore, we next investigated whether URT T_RM_ generated in the presence or absence of local antigen display different tissue localization patterns or exhibit functional differences. We performed adoptive transfer of CD45.2^+^ gp33-specific P14 CD8^+^ T cells into CD45.1^+^ hosts followed by i.n. or i.p. infection with x31-gp33 and assessed T_RM_ localization in the nasal cavity by fluorescence microscopy on day 30–31 post-infection (Fig. 3A). Regardless of local respiratory antigen, URT CD8^+^ T_RM_ displayed similar patterns of localization near epithelial cells within the folds of the nasal turbinates or in glandular-like structures (Fig. 3B). We identified significantly more influenza-specific T_RM_ in equivalently-sized URT tissue sections after i.n. priming compared to i.p. priming (Fig. 3C), supporting our earlier observation that local respiratory antigen results in higher numbers of URT T_RM_ (Fig. 1C). To determine functional capacity, URT T_RM_ were isolated from mice infected with x31 i.n. or i.p. and stimulated with influenza peptides to assess production of TNF-α and IFN-γ (Fig. 3D). There was no significant difference in the frequency of IFN-γ-producing or IFN-γ/TNF-α double-producing influenza-specific URT T_RM_ after i.n. or i.p. infection, although the frequency of TNF-α-producing cells was significantly decreased following i.p. infection (Fig. 3E and F). Overall, these data show that URT T_RM_ formed in the presence or absence of local respiratory antigen have similar cytokine responses.

**Figure 3:**
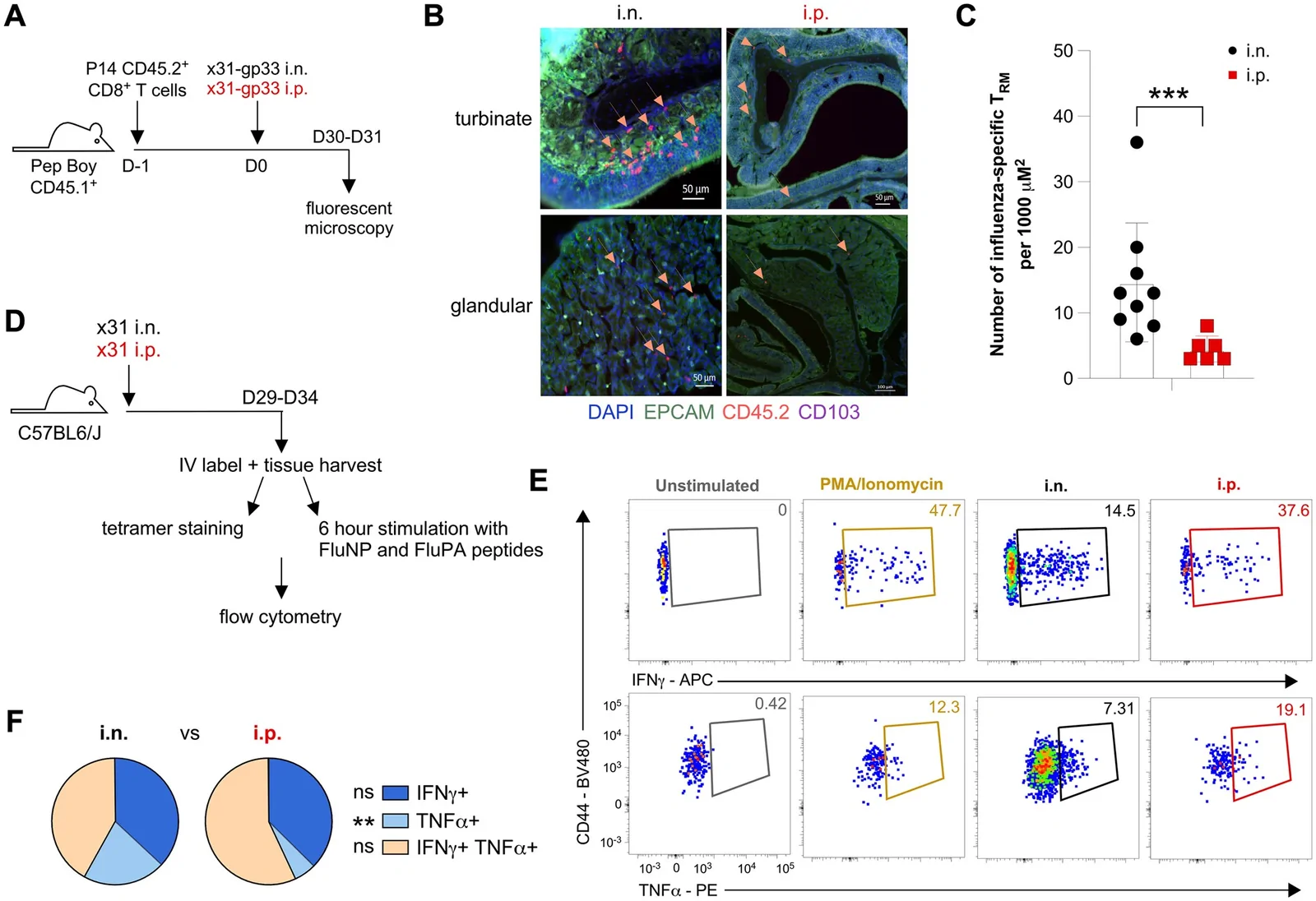
URT CD8^+^ T_RM_ are functionally similar in the presence and absence of local respiratory antigen. (A) Experimental setup to assess localization patterns of influenza-specific URT T_RM_ after i.n. or i.p. priming. (B) Representative images of nasal cavity tissue sections demonstrating influenza-specific T_RM_ localization near respiratory epithelium after i.n. and i.p. priming in turbinate structures and glandular structures. Arrows indicate co-localization of CD45.2 and CD103. (C) Number of influenza-specific T_RM_ per indicated field in nasal cavity tissue sections after i.n. or i.p. priming. (D) Experimental setup to assess functional capacity of influenza-specific URT T_RM_ after i.n. or i.p. priming. (E) Representative intracellular staining for IFN-γ and TNF-α from URT T_RM_ in unstimulated samples, PMA/Ionomycin stimulated samples, i.n. primed mice, and i.p. primed mice. (F) Pie chart illustrating frequency of IFN-γ-producing, TNF-α-producing, or co-producing URT T_RM_ after i.n. priming or i.p. priming. Microscopy images representative of *n* = 10 (i.n.) or *n* = 5 mice (i.p.) (B–C). Quantitative microscopy data represented as mean  ± SD from *n* = 6–9 unique tissue sections per infection condition (C). Quantitative functional data representative of average values from *n* = 9 individual or pooled samples (i.n.) and *n* = 9 pooled samples (i.p.) from *n* = 2 independent replicates (F). Significance determined by Mann–Whitney test (C) or unpaired T test (F). *P* values are as follows: NS 0.1234, *P 0.0332, **P 0.0021, ***P 0.0002, ****P < 0.0001.

### URT T_RM_ have similar transcriptional programs, distinct from LRT T_RM_, regardless of local antigen

While we previously confirmed that URT MPECs can adopt a T_RM_ signature in the absence of local respiratory antigen, we sought to compare URT T_RM_ generated in the presence or absence of local antigen, along with LRT T_RM_, to determine whether antigen encounter during development or localization to different niches in the respiratory tract results in distinct transcriptional programs. To this end, we infected mice i.n. or i.p. with x31 and performed single cell RNA sequencing and TCR sequencing on sorted tissue-resident influenza-specific CD8^+^ T cells at 45 d post-infection. As expected, cells from lung and nasal cavity segregated from the splenic compartment (Fig. 4A). For further analysis of differentially expressed genes (DEGs), we utilized *Itgae* expression to identify CD103^+^ T_RM_ in the respiratory tissues (Fig. 4B). Respiratory tract T_RM_ showed increased expression of several DEGs associated with tissue residency, including *Cxcr6*, *Osgin1*, *Chn2*, *P2rx7*, *Cdh1*, and *B4galnt4*, and decreased expression of *Klf2* (Fig. 4C). A core T_RM_ signature consisting of 27 genes was shared among LRT T_RM_ and URT T_RM_ established in both the presence and absence of local antigen (Fig. 4D). While there were a small number of unique DEGs among URT T_RM_ after i.n. or i.p. infection, there were also few unique DEGs in both groups compared to LRT T_RM_. In contrast, there were a greater number of DEGs unique to LRT T_RM_ when compared to either URT T_RM_ population (Fig. 4D). Taken together, DEG analysis suggests that despite a shared core T_RM_ signature, LRT T_RM_ remain transcriptionally distinct from URT T_RM_. Single cell pathway analysis revealed that all respiratory T_RM_ were enriched for transcripts involved in cell-cell adhesion, response to transforming growth factor beta, and hypoxia pathways (Fig. 4E). URT and LRT T_RM_ signatures after i.n. priming were significantly enriched for genes in the viral response pathway, and LRT T_RM_ signatures were highly enriched for pathways implicated in oxidative stress or hypoxia (Fig. 4E), consistent with previous findings.^25^ Direct comparison of these pathways in URT T_RM_ after i.n. or i.p. priming revealed no significant differences (Fig. 4E), indicating that URT T_RM_ have similar transcriptional profiles in both the presence and absence of local antigen.

**Figure 4:**
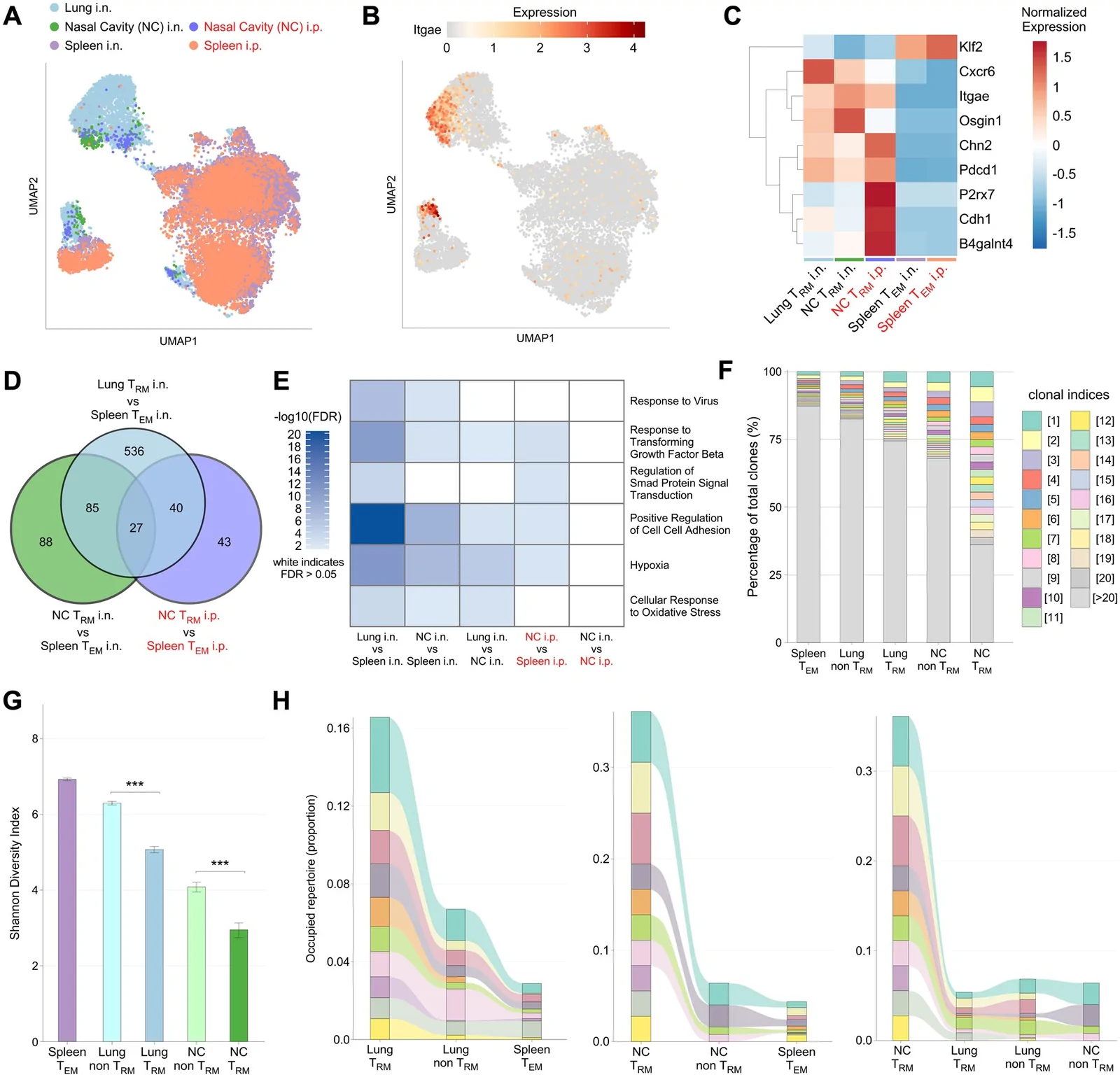
URT CD8^+^ T_RM_ are transcriptionally similar in the presence or absence of local respiratory antigen. (A) UMAP generated from single cell RNA sequencing of total sorted CD8^+^ FluNP^+^ or FluPA^+^ T cells from spleen, URT, and LRT 45 d post i.n. or i.p. priming. (B) *Itgae* expression for identification of respiratory T_RM_, splenic T_EM_, and respiratory non-T_RM_ subsets. (C) Hierarchal clustering heatmap of single cell RNA transcript expression for the indicated differentially expressed genes. (D) Venn diagram showing number of significantly enriched shared or unique DEGs among LRT T_RM_ and URT T_RM_ compared to spleen T_EM_ after i.n. or i.p. priming. (E) Heatmap from single cell pathway analysis (SCPA) showing pathways significantly enriched in indicated comparisons of cell subsets after i.n. or i.p. priming. (F) Clonal index plots showing total number of clones in indicated cell subsets after i.n. priming. Light gray indicates occupied repertoire space for any number of individual clones greater than 20. (G) Shannon diversity index scores in indicated cell subset comparisons after i.n. priming. (H) Clonal sharing plots for top 10 clones showing occupied clonal repertoire and shared clones amongst indicated cell subsets after i.n. priming. Significance determined by bootstrapping and permutation testing (H). *P* values are as follows: *P < 0.05, **P < 0.01, ***P < 0.001, ****P < 0.0001.

We next characterized the respiratory tract TCR repertoire by comparing T_RM_ subsets against splenic T_EM_ or non-T_RM_ cells within the same tissue. As LRT T_RM_ cannot form in the absence of local respiratory antigen (Fig. 1E), our TCR analysis was restricted to i.n. infection only. As expected, clonal indexing revealed the highest degree of clonal diversity in the splenic T_EM_ compartment (Fig. 4F). While there were significant differences in overall TCR diversity among the T_RM_ versus non-T_RM_ within the respiratory compartments (Fig. 4F and G), analysis of the occupied repertoire space indicated that most clones were shared between the compartments, with few unique clones restricted to URT T_RM_ or LRT T_RM_ (Fig. 4H). Overall, these data reflect that while each compartment maintains distinct clonotypic diversity, T_EM_ and T_RM_ likely arose from the same precursor cells during infection, consistent with reports from human studies.^50^

### TGF-β signaling is essential for URT CD8^+^ T_RM_ development only in the absence of local respiratory antigen

Previous studies have demonstrated roles for cytokines such as IL-15, IL-33, and TGF-β in T_RM_ development,^30^^,^^51^^,^^52^ including in the formation of respiratory tract CD8^+^ T_RM_.^20^ However, the potential roles of these cytokines in T_RM_ development in the absence of respiratory antigen have not been explored. To this end, we generated E8i-Cre conditional knockout (cKO) mice to ablate IL15Rα, IL1rL1, or TGF-βR-II in only peripheral CD8^+^ T cells.^53^ Wild-type (WT) control mice and cKO mice were infected i.n. or i.p. with x31 and the frequency of influenza-specific CD8^+^ T_RM_ was assessed by flow cytometry (Fig. 5A and B). Following i.n. infection, ablation of the IL-15 and IL-33 receptors did not impact the frequency of T_RM_ in either LRT and URT (Fig. 5C, Fig. S3A and B). In contrast, ablation of the TGF-β receptor significantly reduced the frequency of T_RM_ in both sites; notably, lack of TGF-β signaling did not fully prevent T_RM_ formation in the URT (Fig. 5C, Fig. S3A and B). Following i.p. infection, we focused on investigating the role of these cytokine receptors in the formation of URT CD8^+^ T_RM_, as LRT T_RM_ are not generated in the absence of local respiratory antigen. Similar to URT CD8^+^ T_RM_ generated following i.n. infection, IL-15 and IL-33 signaling did not impact the frequency of URT T_RM_ formation in the absence of respiratory antigen (Fig. 5D, Fig. S3C). However, in i.p.-primed TGF-β cKO mice, there was a complete loss of URT CD8_+_ T_RM_, despite equivalent numbers of circulating memory CD8^+^ T cells compared to WT (Fig. 5D and G, Fig. S3C), demonstrating that TGF-β signaling is required for T_RM_ formation in this scenario. As expected, there were differences in the frequencies of naïve and memory CD8^+^ T cells in uninfected TGF-βR cKO mice compared to WT controls (Fig. S4A–E); however, this did not impact the expansion of influenza-specific effector CD8^+^ T cells in the tissues and in circulation during the acute stage of infection in the absence of TGF-β signaling (Fig. S4F–K). This differential requirement for TGF-β signaling in URT T_RM_ formation in the presence or absence of local respiratory antigen was confirmed by a direct comparison of the frequency and number of influenza-specific T_RM_ in all three conditions/sites (LRT i.n. priming, URT i.n. priming, URT i.p. priming) in TGF-βRII cKO mice (Fig. 5E). While the ablation of either TGF-βRII or the absence of local respiratory antigen resulted in significant, but partial, reduction in URT CD8^+^ T_RM_, the loss of both TGF-β signaling and the absence of local respiratory antigen completely prevents formation of URT T_RM_ (Fig. 5B and F), demonstrating that TGF-β signaling is essential for URT T_RM_ development in the absence of local antigen.

**Figure 5:**
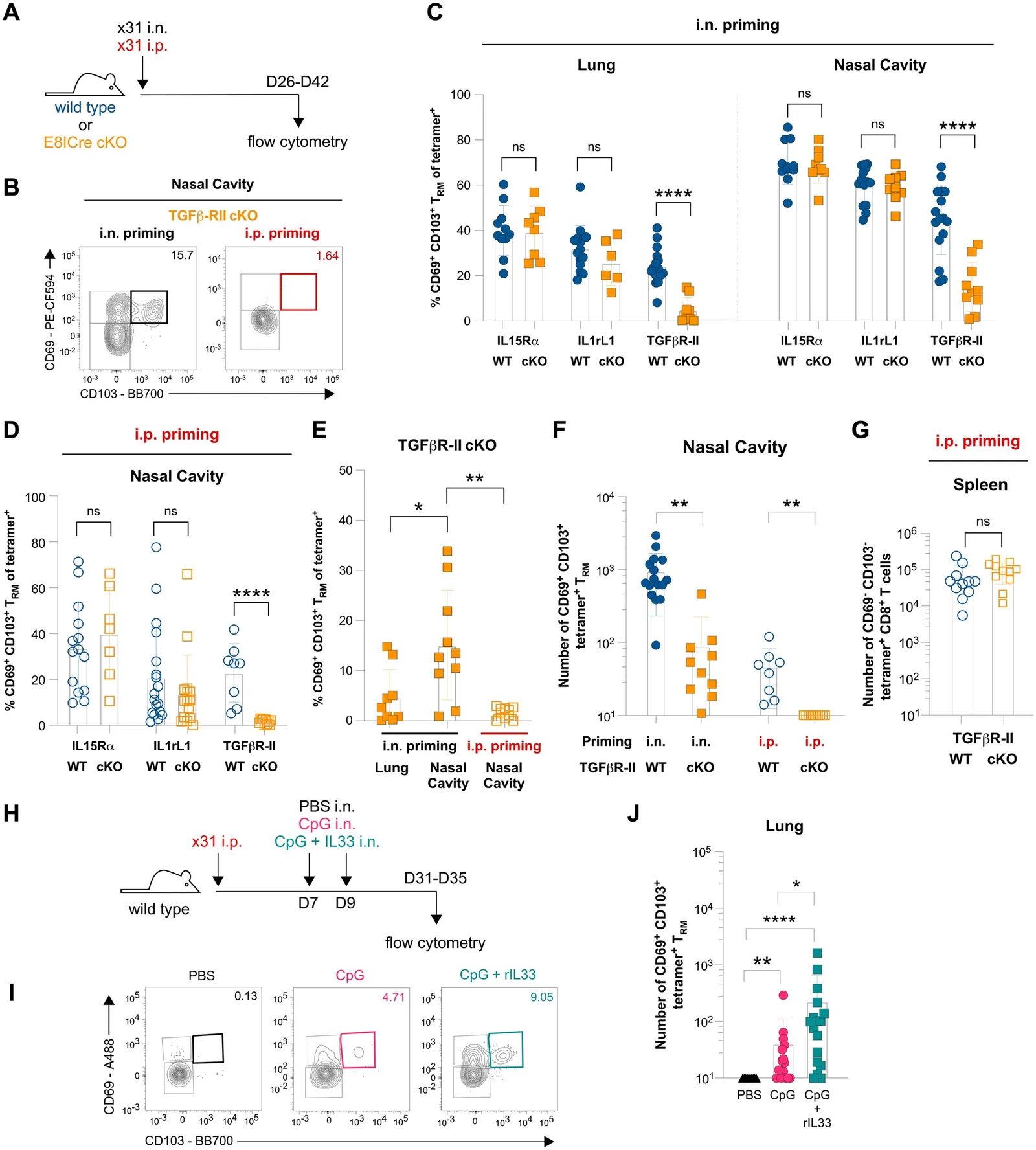
TGF-β signaling is crucial for URT CD8^+^ T_RM_ development in the absence of local antigen. (A) Experimental setup to assess respiratory tract T_RM_ in wild-type (WT) control and conditional IL15R-α, TGF-βR-II, and IL1rL1 knockout (cKO) mice after i.n. and i.p. priming. (B) Representative staining for CD69 and CD103 on influenza-specific URT T_RM_ in TGF-βR-II cKO mice after i.n. and i.p. priming. (C) Frequency of influenza-specific LRT and URT T_RM_ in WT versus cKO mice after i.n. priming. (D) Frequency of influenza-specific URT T_RM_ in WT versus cKO mice after i.p. priming. (E) Frequency of influenza-specific LRT and URT T_RM_ in TGF-βR-II cKO mice after i.n. versus i.p. priming. (F) Absolute number of influenza-specific URT T_RM_ in WT and TGF-βR-II cKO mice after i.n. versus i.p. priming. (G) Absolute number of influenza-specific splenic CD8^+^ T cells in WT and TGF-βR-II cKO mice after i.p. priming. (H) Experimental setup to assess LRT T_RM_ rescue in WT mice after i.p. priming and indicated i.n. dosing. (I) Representative staining for CD69 and CD103 on influenza-specific LRT T_RM_ in WT mice after i.p. priming and indicated i.n. dosing. (J) Absolute number of influenza-specific LRT T_RM_ in WT mice after i.p. priming and indicated i.n. dosing. Data represented as mean ± SD from *n* = 2 to 3 independent experiments with *n* = 8–17 mice per genotype and infection condition (C–F). Absolute cell numbers utilize 10 cell minimum cutoff (F–G, J). Significance determined by unpaired T test (C–G) or Mann–Whitney test (J). *P* values are as follows: NS 0.1234, *P < 0.0332, **P < 0.0021, ***P <0.0002, ****P < 0.000.

Given that TGF-β drives induction of the T_RM_ program through activation of Smad proteins,^54^ we sought to investigate whether other cytokines could compensate for TGF-β signaling to promote LRT T_RM_ development in the absence of local respiratory antigen. For example, signaling through IL-33R can also be mediated by Smad proteins, and there is evidence of shared TGF-β/IL-33 signaling pathways in other settings.^55^^,^^56^ Therefore, we tested whether IL-33 administration could rescue LRT T_RM_ following i.p. infection. Mice were administered CpG only (to control for inflammation alone), or a combination of CpG plus recombinant IL-33 (rIL33) at days 7 and 9 post-infection to draw virus-specific effector CD8^+^ T cells into the respiratory tract (Fig. 5H). While PBS-treated control mice failed to generate LRT T_RM_, treatment with CpG + rIL-33 resulted in a significant increase in the number of influenza-specific LRT T_RM_ over CpG alone (Fig. 5I and J). Together, these data show that IL-33 signaling can rescue LRT T_RM_ development in the absence of local respiratory antigen.

## Discussion

While previous work has elegantly identified site-specific signals that dictate T_RM_ development in various mucosal compartments including the lung,^57–59^ our study defines how the presence of local respiratory antigen alters the cytokine signals required for T_RM_ development in distinct niches of the respiratory tract. We demonstrate that while LRT and URT T_RM_ share a core transcriptional T_RM_ signature, LRT T_RM_ have a transcriptional program that is distinct to the lung environment. In addition, we found that local antigen recognition is a crucial component for LRT T_RM_ development,^20^^,^^32^ but this requirement can be partially overcome by delivery of IL-33. While we do not know why IL-33 can compensate in this scenario, this may suggest that bioactive TGF-β is not readily available in the absence of a local infection in the lung. IL-33 signaling can activate the Smad signaling pathway similarly to TGF-β^56^ thus compensating for a lack of bioactive TGF-β in the absence of local respiratory antigen to drive a T_RM_ program. This pathway may also contribute to lung T_RM_ development in other settings beyond respiratory virus infection, such as asthma, cancer, and transplant, to allow for T_RM_ formation in absence of cognate antigen. Previous work has identified signals important for T_RM_ maintenance in transplant, including local antigen and IL-15 signaling.^60^ Developing a complete understanding of the local antigen and cytokine signaling requirements for LRT T_RM_ development in settings such as transplant could be beneficial for designing interventions to limit T_RM_-mediated rejection or reduce T_RM_-mediated damage in the lung.

In addition to our findings regarding LRT T_RM_ development, this study defines the differential requirement for TGF-β signaling in URT T_RM_ development depending on the presence of local respiratory antigen. The upper respiratory tract is a primary site of initial viral replication following transmission, and URT T_RM_ have previously been implicated as a crucial component of protection against severe disease.^20^^,^^21^ While human studies have demonstrated that virus-specific CD8^+^ T_RM_ in the nasal cavity are established after parenteral vaccination,^31^ the cytokine requirements under this scenario had not been elucidated. Our findings suggest that developing approaches to engage Smad signaling in the absence of local antigen would enhance the development of URT T_RM_ in the absence of respiratory antigen. Indeed, IL-33 administration is being investigated as a vaccine adjuvant candidate to enhance protective antiviral CD8^+^ T cell responses.^61^ While promising, the use of IL-33 as an adjuvant in respiratory virus vaccines will require further investigation into the interplay between local antigen and the Smad/ST2 signaling axis in LRT and URT T_RM_ development.

Finally, while our efforts here focused on segregating the explicit signals needed for LRT versus URT T_RM_ development, it is important to note that inducing T_RM_ in both the URT and LRT is essential for optimal protection against respiratory viruses. Our work shows that formation of T_RM_ in both sites is maximized when local antigen is present, suggesting that optimal protection afforded by T_RM_ would likely be achieved through intranasal vaccination that can deposit respiratory antigen. Indeed, previous studies demonstrate that enhanced protective immunity is achieved through mucosal/intranasal vaccination routes as opposed to parenteral vaccination.^62^^,^^63^ Although correlates of protection for intranasal live attenuated vaccines in humans are often focused on antibody responses, the contribution of T_RM_ to protective immunity has proven to be critical for protection in the absence of virus-specific antibody responses.^21^ While optimal protection can be achieved through mucosal vaccination in mice, exploration of methods to improve parenteral vaccination against respiratory viruses through the development of URT and LRT T_RM_ should be investigated in parallel with mucosal respiratory virus vaccines.

## Supplementary Material

vkag269_Supplementary_Data

## Data Availability

The data that support the findings of this study are available from the corresponding author upon reasonable request and have been deposited into the GEO database under the following accession numbers: GSE327966 (Bulk RNAseq), GSE328000 (scRNAseq).
