# Supplementary material for: Differential requirements for cytokine signaling and local antigen in upper versus lower respiratory tract CD8+ TRM development
Source: J Immunol. 2026 Oct 17;215(10):vkag269. doi: 10.1093/jimmun/vkag269 (PMC13676376; doi:10.1093/jimmun/vkag269)
Supplement: Supplementary file 1 [file vkag269_supplementary_data.zip › V2_Rodgers_et_al_Supplemental_Materials.pdf]

Supplemental Materials

Table S1 Gene sets for gene set enrichment analysis in Fig 2I-J

Up in lung T_{RM}	Down in lung T_{RM}
2900026A02Rik	Duox1
Cdh1	Sell
Fgl2	Gm15987
Xcl1	Fcgr2b
Itgae	Thsd4
Spsb1	Dapl1
Chn2	Gm49066
Styk1	Wfikkn2
Lzts1	Mal
Isg20	Serp2
Tjp1	Plcxd3
Osgin1	Ccdc180
Krt83	Slc18a1
Calca	Pde2a
Irf4	Olfm1
Rgs1	Nsg2
Pde4a	Tdrp
Rgs16	Clec2g
Tnfsf11	Gm35460
B4galnt4	Gm49180
Csf1	Ifngas1
Ikzf2	Sh3bp5
Igflr1	Vipr1
Lyn	Trim30c
Litaf	Atp6v0d2
B3galt5	S1pr5
Ppp2r2c	Gm48249
AA467197	S1pr1
Zswim4	Crlf1
Ier5I	Sned1
Rhoq	Trpm6
Gzma	Gm36199
Rgs2	Sidt1
Pdcd1	Pdgfrl
Tnfrsf9	Fcgrt
Bcl2a1d	Aoah
Isg15	Otoa
Smim3	Hs3st3a1
Itga1	Rflnb
Tesc	Gm17382

Cxcr6	Phex
Icos	Vmn3r57
Uhrf2	A930005H10Rik
Rbpj	Gzmm
Il21r	Cmah
Cmtm7	Acsm3
Itgav	Capn9
	Cyp4f18
	Trim30b

Figure S1

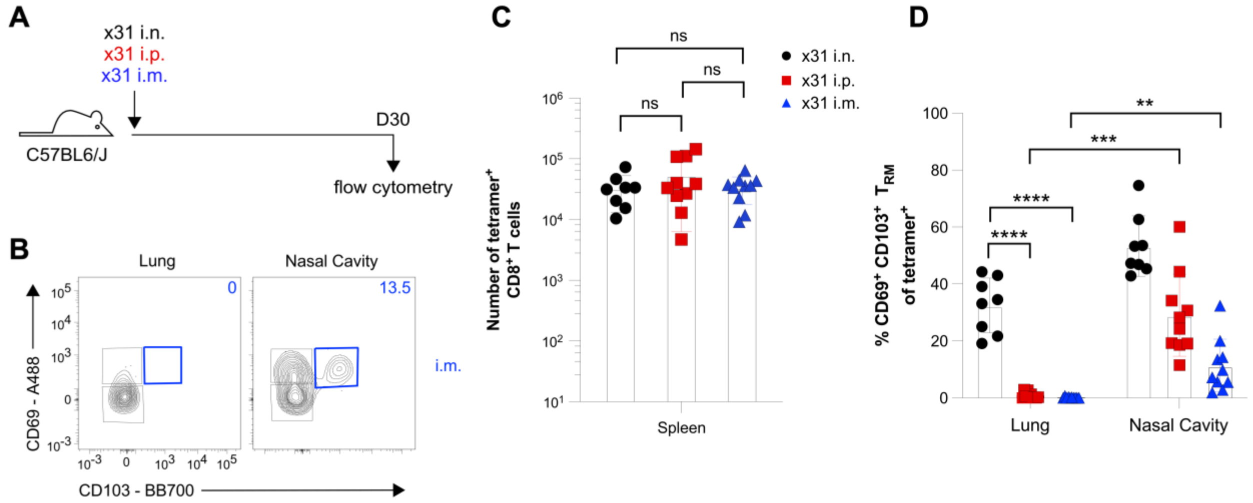

Figure S1. URT CD8^{+} T$_{RM}$ can develop after i.p. or i.m. priming

(A) Experimental setup to assess formation of respiratory tract CD8^{+} T$_{RM}$ after i.n. priming, i.p. priming, or i.m. priming.

(B) Representative staining for T$_{RM}$ markers CD69 and CD103 on influenza-specific CD8^{+} T cells in the LRT and URT after i.m. priming.

(C) Absolute number of influenza-specific CD8^{+} T cells in spleen after i.n. priming, i.p. priming, or i.m. priming.

(D) Frequency of influenza-specific LRT and URT T$_{RM}$ in wild-type mice after i.n., i.p., or i.m. priming.

Data represented as mean ± SD from n=2 independent experiments with n=8-10 mice total per infection condition. Absolute cell numbers utilize 10 cell minimum cutoff (C). Significance determined by unpaired T test (C, D). P values are as follows: NS 0.1234, *P 0.0332, **P 0.0021, ***P 0.0002, ****P < 0.0001.

Alt text: Schematics, flow cytometry plots, and quantitative analysis of respiratory tract tissue-resident T cells in infected wild-type mice, with subfigures labeled from a to d.

Figure S2

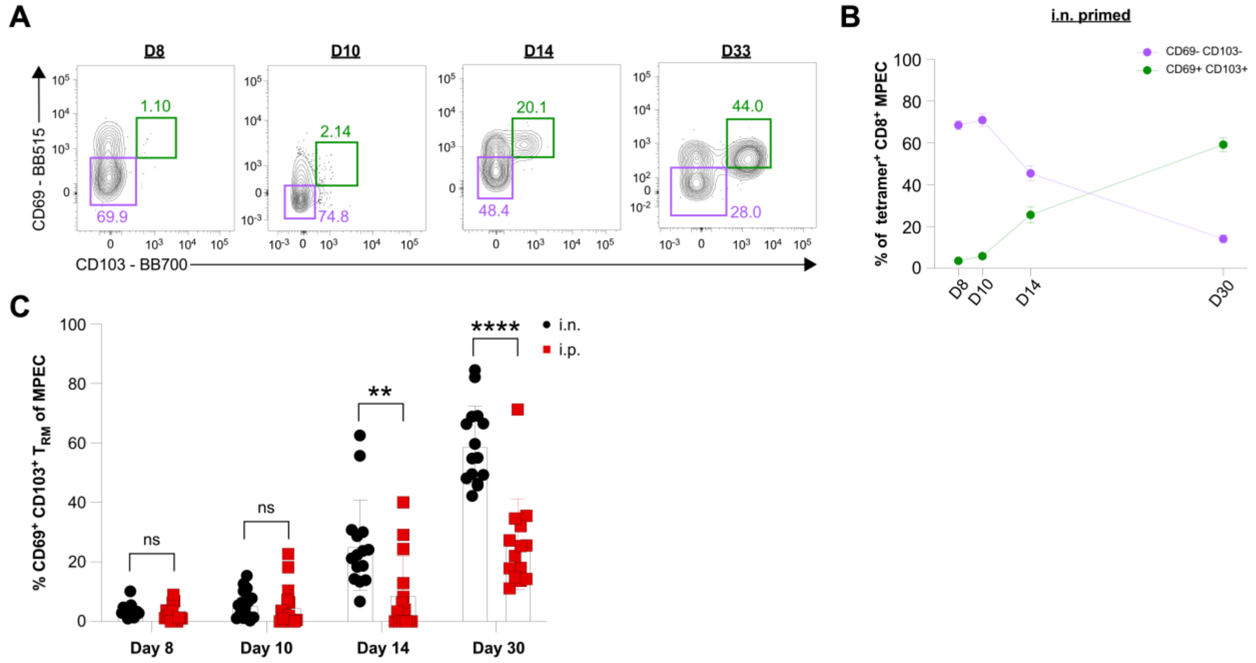

Figure S2. URT $CD8^+$ T_{RM} precursors have similar development kinetics in the presence or absence of local antigen

(A) Representative staining for CD69 and CD103 on influenza-specific URT memory precursor (MPEC) cells over time after i.n. priming.

(B) Frequency of influenza-specific URT MPEC T_{EM} and T_{RM} over time after i.n. priming.

(C) Frequency of URT MPEC T_{RM} over time after i.n. versus i.p. priming.

Data represented as mean ± SEM (B) or mean ± SD (C) from n=3 independent experiments with n=3-15 mice per timepoint and infection condition (B,C). Significance determined by unpaired T test (C). P values are as follows: NS 0.1234, *P 0.0332, **P 0.0021, ***P 0.0002, ****P < 0.0001.

Alt text: Flow cytometry plots and quantitative analysis of developing respiratory tract tissue-resident T cells in infected wild-type mice, with subfigures labeled from a to c.

Figure S3

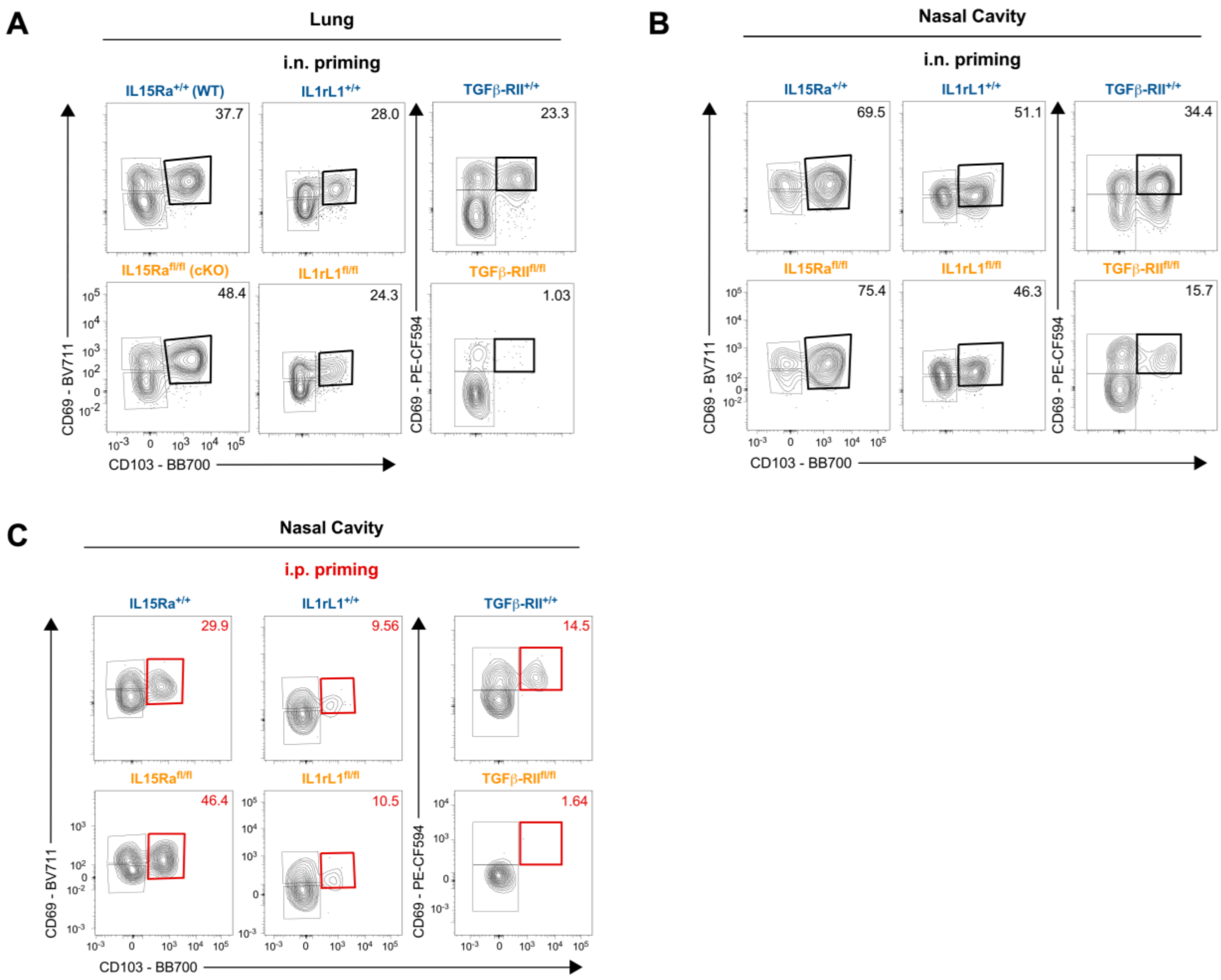

Figure S3. URT and LRT CD8$^+$ T$_{RM}$ have similar requirements for TGF-β signaling in the presence or absence of local antigen

(A) Representative staining for T$_{RM}$ markers CD69 and CD103 on influenza-specific LRT CD8$^+$ T cells in wild-type (WT) control mice and conditional IL15Ra, IL1rL1, and TGFβR-II knockout mice (cKO) after i.n. priming.

(B) Representative staining for T$_{RM}$ markers CD69 and CD103 on influenza-specific URT CD8$^+$ T cells in WT and cKO mice after i.n. priming.

(C) Representative staining for T$_{RM}$ markers CD69 and CD103 on influenza-specific URT CD8$^+$ T cells in WT and cKO mice after i.p. priming.

Data representative of n=2-3 independent experiments with n=8-16 total mice per genotype and infection condition.

Alt text: Flow cytometry plots of respiratory tract tissue-resident cells in infected conditional knockout and wild-type mice, with subfigures labeled from a to c.

Figure S4

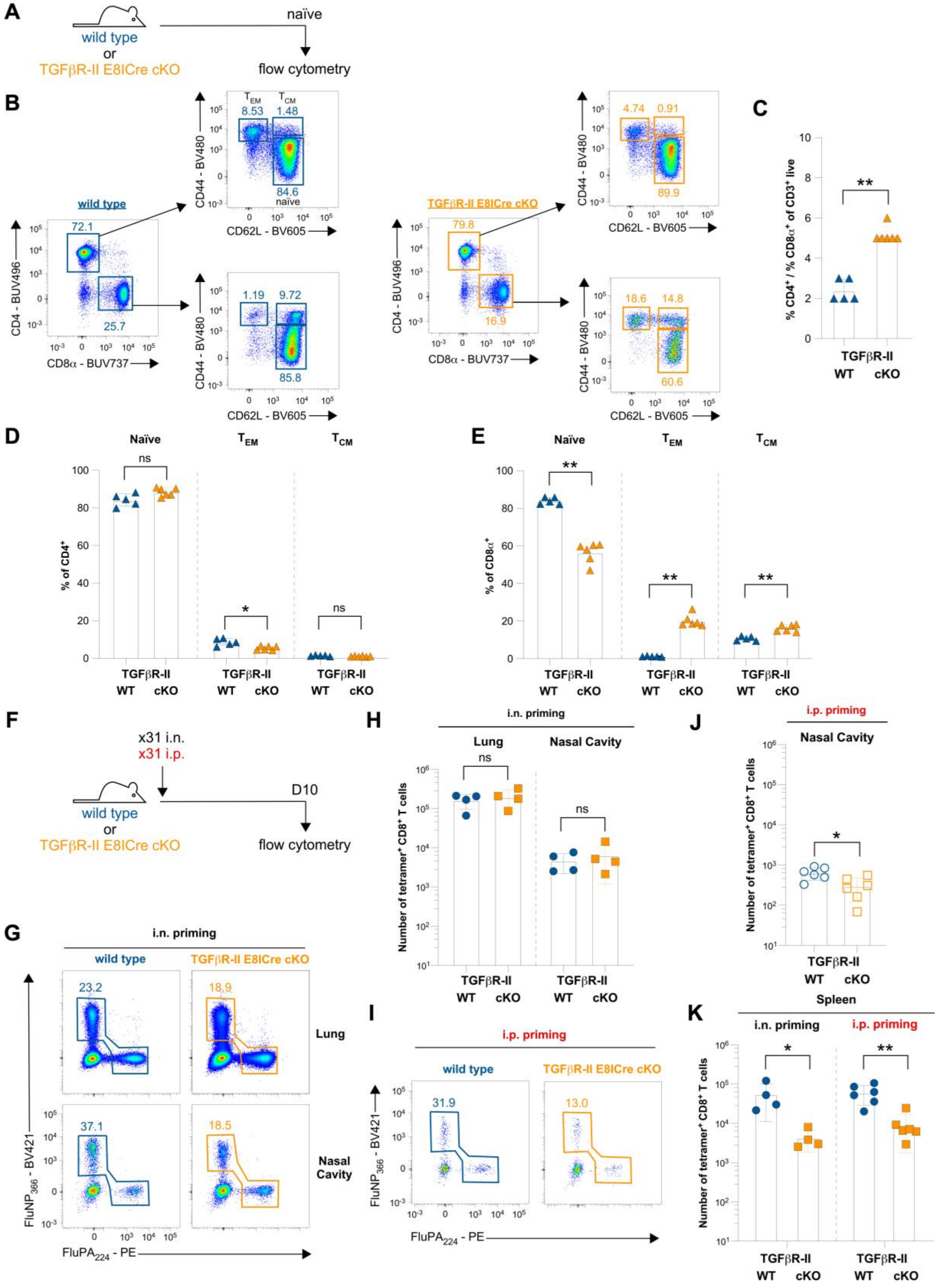

A
wild type
or
TGFβR-II E8ICre cKO
naïve
flow cytometry
B
wild type
TGFβR-II E8ICre cKO
CD4 - BUV496
CD8α - BUV737
CD44 - BV480
CD62L - BV605
T_EM
T_CM
naïve
72.1
25.7
8.53
1.48
84.6
1.19
9.72
85.8
79.8
16.9
4.74
0.91
89.9
18.6
14.8
60.6
C
% CD4+ / % CD8α+ of CD3+ live
**
TGFβR-II
WT
cKO
D
Naïve
T_EM
T_CM
% of CD4+
ns
*
ns
E
% of CD8α+
**
**
**
F
x31 i.n.
x31 i.p.
D10
flow cytometry
G
i.n. priming
Lung
Nasal Cavity
FluNP366 - BV421
FluPA224 - PE
23.2
18.9
37.1
18.5
H
i.n. priming
Lung
Nasal Cavity
Number of tetramer+ CD8+ T cells
ns
ns
I
i.p. priming
31.9
13.0
J
i.p. priming
Nasal Cavity
*
K
Spleen
i.n. priming
i.p. priming
*
**

Figure S4. TGF-β is needed to control $CD8^+$ T cell expansion and aids in formation of URT influenza-specific $CD8^+$ T cells

(A) Experimental setup to assess splenic $CD8^+$ T cells in naïve wild-type (WT) and conditional TGFβR-II knockout (cKO) mice.

(B) Representative staining for CD4 and CD8 splenocytes and identification of naïve ($CD44^{lo}$ $CD62L^+$), effector memory (T_{EM}) ($CD44^{hi}$ $CD62L^-$), and central memory (T_{CM}) ($CD44^{hi}$ $CD62L^+$) circulating T cells in naïve WT and TGFβR-II cKO mice.

(C) Frequency ratio of circulating $CD4^+$ versus $CD8^+$ T cells in naïve WT and TGFβR-II cKO mice.

(D) Frequency of circulating $CD4^+$ naïve T cells, T_{EM}, and T_{CM} in naïve WT and TGFβR-II cKO mice.

(E) Frequency of circulating $CD8^+$ naïve T cells, T_{EM}, and T_{CM} in naïve WT and TGFβR-II cKO mice.

(F) Experimental setup to assess expansion of URT and LRT influenza-specific $CD8^+$ T cells in WT and TGFβR-II cKO mice 10 days post-i.n. versus -i.p. priming.

(G) Representative staining for URT and LRT influenza-specific $CD8^+$ T cells in WT and TGFβR-II cKO mice after i.n. priming. Gated on live IV^- $CD8^+$ $CD44^{hi}$.

(H) Absolute number of LRT and URT influenza-specific $CD8^+$ T cells in WT and TGFβR-II cKO mice after i.n. priming.

(I) Representative staining for URT influenza-specific $CD8^+$ T cells in WT and TGFβR-II cKO mice after i.p. priming. Gated on live IV^- $CD8^+$ $CD44^{hi}$.

(J) Absolute number of URT influenza-specific $CD8^+$ T cells in WT and TGFβR-II cKO mice after i.p. priming.

(K) Absolute number of circulating influenza-specific $CD8^+$ T cells in WT and TGFβR-II cKO mice after i.n. versus i.p. priming.

Data represented as mean ± SD from n=1 independent experiment with n=4-6 mice per genotype and infection condition. Absolute cell numbers utilize 10 cell minimum cutoff (H, J, K). Significance determined by Mann-Whitney test (C-E, H, J, K). P values are as follows: NS 0.1234, *P 0.0332, **P 0.0021, ***P 0.0002, ****P < 0.0001.

Alt text: Schematics, flow cytometry plots, and quantitative analysis of respiratory tract tissue-resident cells in naïve and infected conditional knockout and wild-type mice, with subfigures labeled from a to k.
